# Magnetostrictive Effect of Magnetorheological Elastomers Controlled by Magneto-Mechanical Coupling at the Mesoscopic Scale

**DOI:** 10.3390/polym18030377

**Published:** 2026-01-30

**Authors:** Long Li, Hailong Sun, Yingling Wei, Hongwei Cui, Ruifeng Liu, Hongliang Zou, Weijia Zheng

**Affiliations:** 1 College of Aeronautics and Astronautics, Taiyuan University of Technology, Taiyuan 030024, China; 2 College of Mechanical Engineering, Taiyuan University of Technology, Taiyuan 030024, China; pee770086017@163.com (H.S.);; 3 College of Chemistry and Chemical Engineering, Taiyuan University of Technology, Taiyuan 030024, China; 4 School of Mechatronics Engineering, Harbin Institute of Technology, Harbin 150001, China

**Keywords:** magnetorheological elastomers, magneto-mechanical coupling, RVE, magnetostriction, mesoscopic scale

## Abstract

Magnetorheological elastomers (MREs) have attracted considerable attention in high-precision sensing and intelligent control due to their responsive sensitivity. The magnetostrictive properties of MREs excited by magneto-mechanical coupling at the mesoscopic scale show broad application potential but have not yet been fully elucidated. In this study, the magnetostrictive properties were investigated at the mesoscopic scale through theoretical modeling, numerical simulation and experimental research. A correction factor was introduced to address the limitations of conventional magnetic dipole theory under near-field conditions, thereby providing a rational theoretical explanation of magnetostrictive behavior. Visualization analysis was performed using the finite element method (FEM). Subsequently, MREs were prepared under various solidified magnetic fields, and their performance was validated through scanning electron microscopy (SEM) and a laser displacement sensor. The results demonstrated that magnetostriction is determined by the relative angle between the particle chain and the magnetic field direction. The linearity of the particle chain was found to be positively correlated with magnetostriction. The maximum theoretical and experimental magnetostrictive elongations reached 0.9% and 0.565%, respectively, while the maximum theoretical and experimental magnetostrictive compression reached 2.77% and 1.81%, respectively. This work provides significant scientific insights into the magneto-mechanical energy conversion mechanism and contributes to the development of magnetostrictive instruments.

## 1. Introduction

MREs are a class of intelligent materials capable of responding to external stimuli, exhibiting mechanical properties with reversible regulation, and enabling precise control of macroscopic responses [1,2,3]. Their magnetostrictive properties have been applied in high-precision force sensing [4], braking drives, and intelligent bridge control systems [5], making them a frontier of both academic research and engineering practice. A comprehensive understanding of the magnetostrictive behavior of MREs can unlock further application potential. However, the magneto-mechanical excitation mechanism and the critical influencing factors at the mesoscopic scale remain insufficiently understood, posing an urgent scientific challenge.

Numerous studies have examined the magnetostrictive mechanisms of MREs from various perspectives. Yuan Feiyang [6] and Danas K. [7] interpreted the mechanism from theoretical and experimental viewpoints, respectively. Yuan proposed that the linearity of the particle chain governs magnetostriction: highly linear chains result in magnetostrictive compression, whereas less linear chains result in elongation. Danas confirmed that isotropic MREs generally exhibit magnetostrictive elongation along the magnetic field direction. In contrast, Zhou GY [8] experimentally demonstrated that anisotropic MREs may undergo magnetostrictive compression under a uniform magnetic field. Gong Xinglong [9], Oleg V. Stolbov [10], and others showed that uniformly distributed particle chains lead to compressive magnetostriction under an external magnetic field, whereas agglomerated chain structures result in elongation, thus partially explaining the differences observed in prior experiments.

In 2025, Eric M. Stewart [11] of the Department of Mechanical Engineering, Massachusetts Institute of Technology, conducted in-depth research on the magnetostriction of soft magnetorheological elastomer (s-MRE) through theory and simulation. The study revealed that the fundamental driving force for deformation at the s-MRE/air interface is the difference between the Maxwell stress in air $T^{\mathrm{air}\mathrm{maxw}}$ and the combined magnetic stress ($T^{\mathrm{maxw}}+T^{\mathrm{mag}}$) in the solid—this difference constitutes the magnetostriction of the s-MRE. Lefevre, Victor [12], through constitutive modeling and experimental tests, found that MREs filled with ferrofluid particles exhibit magnetostrictive capabilities far superior to those filled with iron particles. The results also highlighted a strong heterogeneity in the deformation and magnetic fields within the specimens, strongly influenced by specimen shape, particularly in iron-particle-based MREs.

Building on foundational theories of magnetic particle interactions within MREs [13,14,15], further investigations have been carried out to clarify the mechanisms of magnetostriction under various conditions. Eraslan et al. [16] studied the representative volume element (RVE) size of MREs and determined the lower limit for valid mesoscopic analysis. Eric M. Stewart et al. [11] developed a set-dependent magnetostrictive response model capable of simulating cylindrical s-MRE specimens. Zhang Qiushu et al. [17,18] simulated the magnetostrictive stress of PDMS-based (Polydimethylsiloxane, PDMS) MRE films, demonstrating that stress is strongly dependent on the nanoparticle concentration and microstructural features. Oleg V. Stolbov et al. [10] simulated the s-MRE, observing only elongation in untreated samples. They attributed this to the prevalence of multi-particle clusters in real materials. In the latest research, Shengwei Feng conducted in-depth research on the dipole model, and pointed out the limitations of the classical dipole theory only when the particle spacing is large (greater than three times the particle size) and the system is thin [19].

The above literature highlights the progress made in understanding the magnetostrictive mechanisms of MREs and their practical applications. However, inconsistencies remain in interpreting magnetostrictive behavior from different perspectives. While significant advances have been achieved in macroscopic characterization and phenomenological modeling, systematic insights into the mesoscopic magneto-mechanical coupling mechanism are still lacking.

In this study, a magneto-mechanical coupling constitutive model for carbonyl iron powder (CIP) particles was developed. The mesoscopic origin of the magnetostrictive effect in MREs was determined. By correlating cross-scale simulations-from the reorientation of the mesoscopic-scale magnetic dipoles to macroscopic mechanical responses - microscopic magnetic events were linked to observed material behavior. Using a multi-physical field coupling model within the framework of mesoscopic mechanics, combined with RVE-based simulations, a full-chain visualization was achieved—from the mesoscopic simulation to the macroscopic strain response. This clarified both the mechanisms of magnetostriction in MREs and the laws governing their dynamic regulation.

## 2. Study on Micromechanics of MREs

Compared with traditional magnetostrictive materials (such as the Terfenol-D alloy or ferrite ceramics), magnetorheological elastomers (MREs) exhibit more significant magnetostrictive properties. Due to the intrinsic diamagnetism of the rubber matrix, its coupling with the magnetic field is negligible. Therefore, the magnetostrictive effect is mainly induced by the interaction between magnetic particles and external magnetic fields. Based on this physical essence, this study conducted in-depth research on the magneto-mechanical behavior of magnetic particles.

### 2.1. Magnetic Dipole Force Between Magnetic Particles

Under an external magnetic field, according to the magnetic dipole approximation framework, the magnetization behavior of CIP particles can be represented by a rigid dipole model with an equivalent magnetic moment. To construct an analytical magneto-mechanical coupling constitutive relationship, the following assumptions were made: all CIP particles are ideal spheres with a radius *R* and the spherical center distance between two particles is *d*; the demagnetization effect cannot be ignored; the magnetization intensity is uniformly distributed [20]; and the ratio of the center spacing to the radius satisfies d/R≥2.2 [6].

(1) Derivation of magnetic dipole moment of a single particle

**Figure 1:**
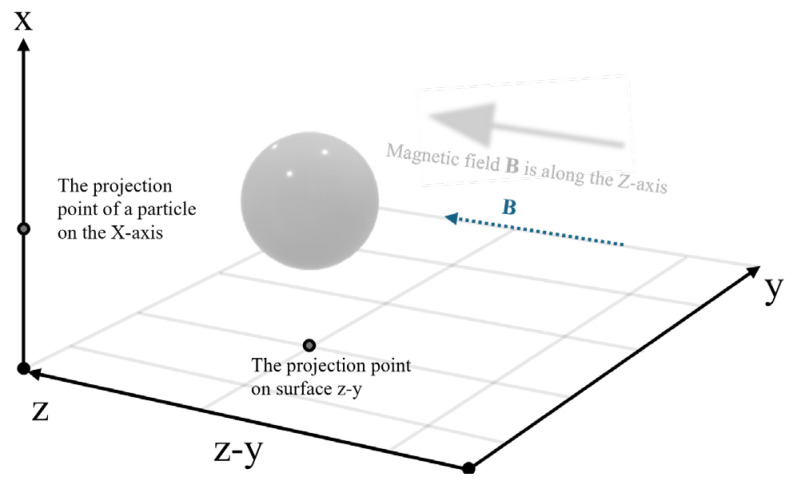
Single magnetic dipole.

Suppose that the magnetic flux density of the applied magnetic field is $B=B\hat{z}$ and the direction is positive along the z-axis of thespatial rectangular coordinate system, as shown in Figure 1. Combined with the vacuum permeability $\mu_{0}$, the external magnetic field strength can be obtained as $H_{0}=B/\mu_{0}$, and the demagnetization field is $H_{d}=-NM$ (the demagnetization factor of the sphere is 1/3, M denotes the magnetization of particles). Therefore, the total magnetic field in the particle can be expressed as $H_{in}=H_{0}+H_{d}$, and the magnetic dipole moment **m** can be expressed as:

(1) $$m=\frac{4\pi R^{3}\left(\mu_{r}-1\right)B}{\mu_{0}\left(\mu_{r}+2\right)}\hat{z}$$

Here μ is the absolute permeability and $\mu_{r}$ is the relative permeability of CIP.

(2) The general expression of the interaction force between magnetic dipoles

Because the interaction force between adjacent magnetic dipoles between different particle chains is weak and can be ignored, two adjacent particles in the same particle chain are taken as the research object. The line connecting the two particle centers is parallel to the direction of the magnetic induction line. Based on the potential energy of a meagnetic dipole (in an external magnetic field), the interaction potential energy U and dipole–dipole force $F_{\mathrm{dipole}}$ of two magnetic dipoles $m_{1}$ and $m_{2}$ can be expressed as

(2) $$U=-m_{2}\cdot B_{1}\;\;\;\;\;F_{\mathrm{dipole}}=\frac{3\mu_{0}}{4\pi }\nabla \left(\frac{m_{1}\cdot m_{2}}{r^{3}}-\frac{3\left(m_{1}\cdot r\right)\left(m_{2}\cdot r\right)}{r^{5}}\right)$$

where r is the variable distance vector between the field point and the source point, $B_{1}$ is the magnetic field generated by $m_{1}$ at $m_{2}$, and $B_{1}$ can be calculated by far-field magnetic field of a dipole. When the two dipoles are arranged collinearly along the z-axis, since the $m_{1}=m_{2}=m\hat{z}$ interaction force is a negative gradient of potential energy, the interaction force F as the negative gradient of potential energy can be expressed as

(3) $$F=-\nabla U=-\frac{\partial }{\partial d}\left(-\frac{\mu_{0}m_{1}m_{2}}{2\pi d^{3}}\right)\hat{z}=-\frac{3\mu_{0}m^{2}}{2\pi d^{4}}\hat{z}$$

The negative sign in the formula indicates that the interaction force is attractive. It can be proved that when the direction of the external magnetic field is parallel to the direction of the particle chain, the magnetic dipole force between the particles is attractive, which provides a strong theoretical basis for the subsequent magnetostrictive compression.

The classical magnetic dipole force formula relies on the point dipole assumption, in which the magnetic dipole moment is concentrated at a geometric point. This assumption remains valid under the condition that d≫R. However, when the center-to-center distance *d* between particles becomes comparable to the particle radius *R* (e.g., d=2.5R), the point dipole assumption breaks down. As two particles approach each other, the actual magnetic interaction takes place between their nearest surfaces rather than between their spherical centers. At such small separations, the physical meaning of the minimum action distance principle and the surface-to-surface spacing must be considered, as this spacing serves as the key parameter governing the interaction strength. Direct use of the center-to-center distance d would lead to an overestimation of the interaction force. In this study, an equivalent spacing correction factor η was introduced to refine the classical formulation. Its physical meaning lies in mapping the actual center-to-center distance d to a larger effective distance $d_{eff}$, thereby reflecting the physical reality that the interaction is governed not by the center-to-center separation, but by the spacing between the nearest surfaces. The equivalent spacing is defined as follows:

(4) $$d_{eff}=\eta d$$

Based on the idea of the ‘center–center’ effect, η is a dimensionless correction factor with value. When *d* ≫ *R*, *η*→1, the model degenerates to the classical point dipole case. When *d*→2*R*, due to the strong near-field interaction and surface effect, η will be less than 1, so that the corrected force $F_{\mathrm{modified}}\propto 1/{\left(\eta d\right)}^{4}$ can reproduce the significant enhancement of the interaction force in the real situation, which is qualitatively consistent with the accurate multipole theory calculation results [15]. For different MREs, η can be determined by a systematic finite element numerical simulation or comparison with control experimental data, so that it is directly related to the measurable structural property of the geometric arrangement (*d*/*R*) of the particles. 

By substituting the modified equivalent spacing $d_{eff}$ into the classical force Equation (3), the modified magnetic interaction force can be obtained. Sorting can be obtained, and the expression of the final magnetic dipole interaction force between two CIP spherical particles is obtained:

(5) $$F_{\mathrm{mag}}=-\frac{24\pi R^{6}{\left(\mu_{r}-1\right)}^{2}B^{2}}{\mu_{0}\eta^{4}d^{4}{\left(\mu_{r}+2\right)}^{2}}\hat{z}$$

$\eta =\eta \left(d/R\right)$ quantitatively characterizes the transition of the interaction from the ‘center–center’ mode in the far field to the ‘surface–surface’ mode in the near field, and the theoretical assumptions of the model are satisfied. This provides a solid theoretical foundation for subsequent magnetostriction testing and simulation of MREs.

Thus, introducing the equivalent spacing correction factor into the classical magnetic dipole force theory provides a novel approach for accurately calculating dipole forces between near-field micron-sized magnetic particles.

### 2.2. Particle Magnetic Field Force Generated by External Magnetic Field Magnetization

In a uniform magnetic field, the magnetic field spatial gradient is zero $\left(\nabla B=0\right)$ and the net force on the dipole is zero. Thus, in a uniform field, the magnetostriction of MREs originates from inter-particle magnetic dipole forces, and not from the direct action of the external magnetic field.

However, in practice, even within a microscale region of 5 μm, the applied field $\left(\nabla B\right)$ exhibits a small gradient. Therefore, the net force is not strictly zero. If the applied field has a gradient (i.e., ∇B≠0), the force acting on a magnetized particle is expressed as:

(6) $$F_{2}=\frac{4\pi \chi_{m}R^{3}}{3\mu_{0}}\nabla \left(B^{2}\right)$$

Here $\chi_{m}$ represents the magnetic susceptibility of CIP particles. The force is proportional to the square of the magnetic field gradient, and its direction is aligned with the direction of increasing magnetic field strength.

### 2.3. Micromechanics and Deformation of PDMS

Because the particles are confined within the elastomer matrix, and the magnetic dipole interaction force is non-zero under the above theoretical model, differential displacement tendencies inevitably arise among particle groups. This displacement trend excites the elastic restoring force system in the superelastic PDMS matrix, thereby generating a continuous observable deformation field. The rubber reaction force of each ball needs to balance the external attraction *F*. According to the static balance, the reaction force of each ball is *F*, and the direction is opposite to the attraction. Therefore, the reaction force of rubber to each ball is *F*. When a single rigid particle undergoes a displacement *u* in an infinite elastic medium, the reaction force is expressed as:

(7) $$F_{self}=\frac{6\pi RE}{\left(1+\nu \right)}\cdot \frac{1-\nu }{1-2\nu }\cdot u$$

where E is Young’s modulus and ν is Poisson’s ratio. The additional displacement caused by another particle at distance d can be calculated using the elastic Green’s function. The displacement component along the connection direction can be expressed as:

(8) $$u_{\mathrm{coupling}}=\frac{F}{4\pi Gd}=\frac{F\left(1+\nu \right)}{2\pi Ed}$$

Here $G=E/\left(2\left(1+\nu \right)\right)$ is the shear modulus. Finally, the total displacement equation can be derived. From the symmetry, the displacement of the two particles is u, which satisfies the following equation:

(9) $$u=\frac{F\left(1+\nu \right)\left[2d\left(1-2\nu \right)+3R\left(1-\nu \right)\right]}{6\pi ERd\left(1-\nu \right)}$$

It can be seen from the above that the reduction in the inter-particle distance is 2u, and the local strain field is determined by the displacement gradient $\varepsilon_{ij}=\left(u_{i,j}+u_{j,i}\right)/2$. This theoretical model, which combines Green’s function and the superposition principle of the elastic mechanics, is well-suited for a multi-body interaction analysis at the microscale level. It provides a rigorous theoretical framework for studying the micromechanical behavior of elastically encapsulated particle systems, and lays a solid foundation for subsequent simulation and experimental investigations.

### 2.4. Force Analysis and Movement Trend of CIP with Non-Ideal Particle Chain Structure

In this part, the force of particle C is studied in depth. The total magnetic field $B_{\mathrm{total}}$ at particle C is the vector sum of $B_{A}$ and $B_{B}$. It can be seen from the vector symmetry that the z components of $B_{A}$ and $B_{B}$ cancel each other to 0, and the y components are superimposed. Since $m_{C}=\alpha B\hat{y}$, only the gradient of $B_{\mathrm{total}}$ needs to be calculated. Let $B_{z}=\left(\mu_{0}\alpha B/2\pi \right)\cdot \left[\left(2h_{z}^{2}-h_{y}^{2}\right)/{\left(h_{y}^{2}+h_{z}^{2}\right)}^{5/2}\right]$; then, the force in the y direction is:

(10) $$F_{y}=\frac{3\mu_{0}\alpha^{2}B^{2}h_{y}\left(h_{y}^{2}-4h_{z}^{2}\right)}{2\pi {\left(h_{y}^{2}+h_{z}^{2}\right)}^{7/2}}$$

Among them, α is the comprehensive proportional coefficient, which connects the magnetic dipole moment m generated by the magnetization of the ball by the external magnetic field with the external magnetic field B, which is defined as $\alpha =\left(4\pi R^{3}/3\right)\cdot \left[\chi_{m}/\mu_{0}\left(1+\chi_{m}\right)\right]$.

From Equation (10), the force direction and movement trend of the particles can be inferred. When $h_{y}^{2}<4h_{z}^{2}$,$F_{y}<0$; that is, particle C is subjected to the force in the negative direction along the y-axis, resulting in a movement trend in the direction of h reduction. When $h_{y}^{2}>4h_{z}^{2}$,$F_{y}>0$, but because $h_{y}\leq 3.5\;\mu m$, this situation does not hold. However, it can be seen from the symmetry that when the three particles A, B and C are in the complete particles, the particles A and B are subjected to the force along the positive direction of the y-axis, and will also produce a movement trend towards the direction of h reduction. Therefore, within the allowable h range, the particle C is always subjected to the force along the negative direction of the y-axis, resulting in a movement trend towards the direction of the particle chain linearity improvement. When $h_{y}$ approaches the allowable maximum $\left(h_{y}\to 3.5\;\mu m\right)$, the denominator ${\left(h_{y}^{2}+h_{z}^{2}\right)}^{7/2}$ growth plays a dominant role, and the force $F_{y}$ attenuates significantly. Specifically, when $h_{y}\approx 3.5\;\mu m$ and $h_{z}=5\;\mu m$, the force $F_{y}$ can be expressed as follows:

(11) $$F_{y}\propto \frac{h}{{\left(h^{2}+d^{2}\right)}^{7/2}}\sim \frac{h}{d^{7}}(h\ll d)$$

Here, the equivalent spacing correction factor ϑ is introduced to correct the far-field condition in classical theory [21], and $h_{y}$ is replaced by the equivalent spacing ${h^{\prime }}_{y}=\vartheta \cdot h_{y}$, where ϑ satisfies $\vartheta =1+\alpha {\left(R/h_{y}\right)}^{n}$ and α and n are empirical constants. The confirmation method of ϑ is similar to the above η confirmation method. By modifying Formulas (10) and (11), the force of particle C in the magnetic field can be modified as follows:

(12) $$F_{y}=\nabla \left(m_{C}\cdot {H^{\prime }}_{total}\right)\propto \frac{h}{{\left[{\left(\vartheta h\right)}^{2}+d^{2}\right]}^{5/2}}\cdot \left[1+\varpi {\left(\frac{R}{h}\right)}^{m}\right]$$

ϖ and $t_{1}$ are adjustment parameters to further optimize the near-field force attenuation trend. When $h_{y}\to 2.2R$, ϑ increases significantly, the equivalent spacing ${h^{\prime }}_{y}$ increases, and the denominator growth rate decreases, which makes the actual force ratio larger than that predicted by the classical model, and the characteristics of the near-field interaction are compounded. When $h_{y}\gg R$,ϑ≈1, the correction term can be ignored, and the classical theory can be regressed. In this study, ϑ was introduced to adjust the effective spacing, which compensates for the shortcomings of the classical model under near-field conditions and is more suitable for practical application conditions.

When the results are extended to the whole chain, for the particles at the end of the chain, the force is not zero along the z-axis direction, but there is an attraction with the adjacent particles along the z-axis direction, which will produce a movement trend close to the adjacent particles. By analogy, the equilibrium state of all connected particles will be broken so that the magnetostrictive compression characteristics of all connected particles will appear. Equation (12) shows that with decreasing particle linearity, the magnetostrictive compression characteristics of MREs decrease rapidly.

### 2.5. Magnetostrictive Elongations

Under the action of specific external magnetic field conditions, MREs can exhibit a magnetostrictive elongation phenomenon. In order to better understand the phenomenon of MREs, this study deduced the theoretical model of magnetostriction of MREs based on the modified magnetic dipole theory. As shown in Figure 1, it is assumed that the magnetic field is positive along the y-axis, and the two adjacent particle centers in the particle chain are connected along the z-axis. Consider two spherical particles with magnetic dipole moments $m_{1}$ and $m_{2}$ aligned along the y-axis (parallel to the external field B). Based on the magnetic dipole interaction energy formula, the force is determined by the gradient of the potential energy:

(13) $$F=-\frac{dU}{dd}=\frac{3\mu_{0}m_{1}m_{2}}{4\pi d^{4}}$$

The force acts along the line connecting the particle centers, manifesting as a repulsive force. Thus, when the external magnetic field is oriented perpendicular to the particle chain, the inter-particle magnetic dipole force is repulsive, providing a strong theoretical basis for magnetostrictive elongation.

**Figure 2:**
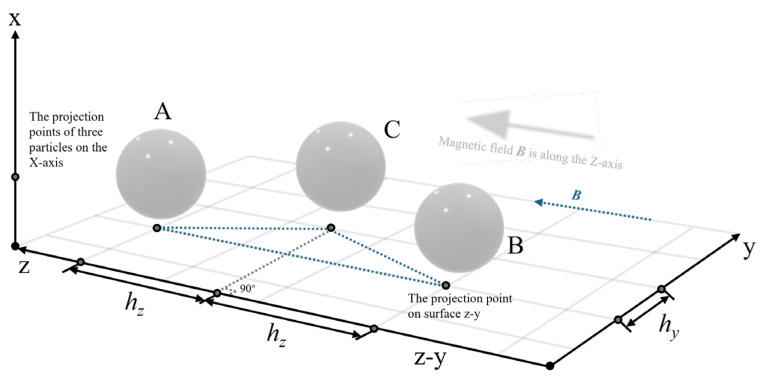
Stress of non-collinear particles.

In order to extend this result to the general case as shown in Figure 2. To account for near-field effects, the equivalent spacing correction factor ξ is also introduced. The spherical center distance is $r=\sqrt{h_{y}^{2}+h_{z}^{2}}$ and the corrected effective horizontal spacing is $r^{\prime }=\sqrt{{\left(\xi h_{y}\right)}^{2}+h_{z}^{2}}$, so the repulsive force of the spherical center connection based on $r^{\prime }$ correction can be expressed as:

(14) $$F^{\prime }=\frac{3\mu_{0}m^{2}}{4\pi {\left[{\left(\xi h_{y}\right)}^{2}+h_{z}^{2}\right]}^{2}}$$

The repulsive force predicted by the corrected model (14) is smaller than that of the classical model. This is because $r^{\prime }>r$ makes the results more in line with the characteristics of faster attenuation of the actual force under near-field conditions. 

For the particle chain shown in Figure 2, particle C is subjected only to force along the y direction. Considering near-field higher-order corrections, an additional correction factor was introduced. After the introduction of additional terms, it can be expressed as

(15) $${F^{\prime }}_{y}=F_{y}\cdot \left[1+\beta {\left(\frac{R}{h}\right)}^{t_{2}}\right]$$

In the formula, β and $t_{2}$ are adjustment parameters used to match the attenuation trend of near-field forces. The confirmation method of the two parameters is similar to the above η confirmation method. Due to the increased equivalent spacing, the vertical repulsive force (14) is reduced compared with the classical model, thereby slowing the longitudinal separation of the particle chain. Under near-field conditions, however, the corrected expression (14) increases significantly, allowing particle C to move more readily in the direction of decreasing particle chain linearity, thereby enhancing the magnetostrictive elongation phenomenon. 

As h approaches its maximum value, the denominator growth is partially offset by ξ, leading to slower force attenuation, which better reflects the weak interaction characteristics of MRE particle chains. Extending this analysis to the entire chain reveals that for end particles, the force along the z-axis is non-zero and repulsive relative to adjacent particles. This induces a movement trend away from neighbors, breaking the equilibrium of the particle chain and giving rise to overall magnetostrictive elongation. As the inter-particle spacing *h* increases, the magnetostrictive elongation characteristics of MREs decrease rapidly.

## 3. Finite Element Simulation of Magnetostrictive Mechanism of MREs

In this section, the RVE model of MREs is established using FEM to achieve a coupled observation of the magnetization behavior of CIP particles and the dipole–dipole interaction mechanism under an applied magnetic field. The microscopic mechanism underlying the dual magnetostrictive modes is systematically investigated. Furthermore, the influence of multiple coupling factors—such as the magnetic field strength and particle chain linearity—on the macroscopic magnetostrictive response is revealed [22].

### 3.1. Magnetization Simulation of CIP Particles Under Magnetic Field

**Figure 3:**
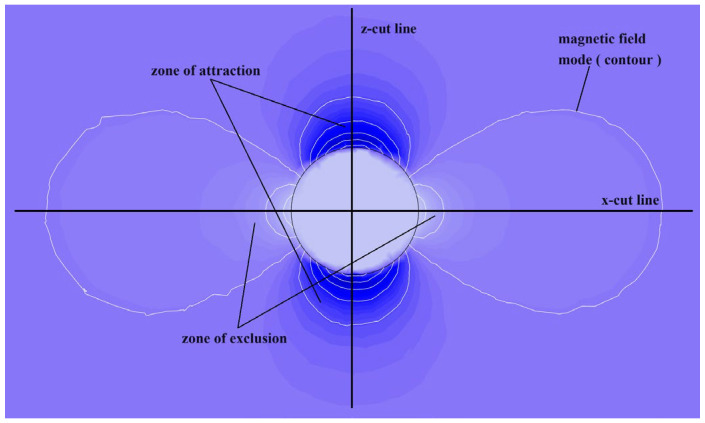
Magnetization of CIP particle in magnetic field.

A magnetic–mechanical coupling analysis model of CIP particles was constructed. In a three-dimensional spatial coordinate system, an ideal magnetic environment with a reduced uniform background magnetic field was established. A magnetic induction intensity field of 0-1 T was applied along the spatial gradient direction. CIP particles with a radius *R*$\left(R=2\;\mu m\right)$ were positioned at the origin of the coordinate system. A multi-physical field coupling algorithm was employed to investigate (i) the spatial distribution characteristics of particle magnetization in a uniform magnetic field, (ii) the dynamic response between saturation magnetization and the applied magnetic field, and (iii) the polarization effects of the magnetic domain structure at the particle ends. The simulation model comprehensively incorporated the Maxwell stress tensor, the magnetostrictive constitutive equation, and the nonlinear magnetization characteristics of ferromagnetic materials, providing computational support for elucidating the magnetic–mechanical conversion mechanism of MREs. When the applied magnetic field was oriented along the z-axis, the magnetization state of the particles was obtained as shown in Figure 3.

According to the analysis of magnetization dynamics and magnetic domain reconstruction theory, it was observed that under the influence of an external magnetic field, magnetic particles exhibit polar aggregation along the axial direction of the field, thereby forming a characteristic magnetic attraction region. In contrast, on both lateral sides (normal direction), magnetic dipole interactions generate a repulsive effect, producing symmetrically distributed repulsive regions (Figure 3).

**Figure 4:**
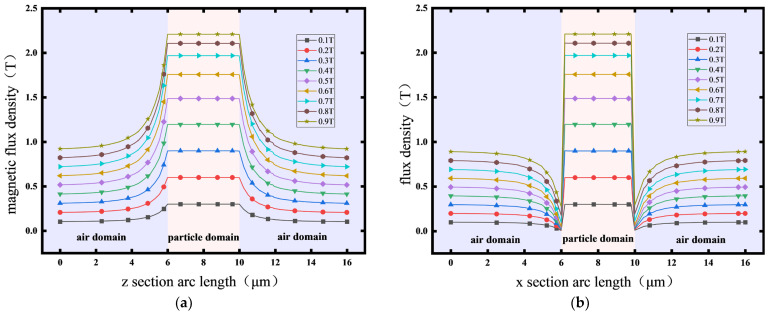
Magnetic field distribution map of CIP particles. (**a**) Magnetic field distribution along the z-line; (**b**) magnetic field distribution along the x-line.

To quantitatively characterize the anisotropic gradient distribution of the magnetic field, the characteristic cross-section along the x-z coordinate system was selected. The gradient distribution of the magnetic field intensity and magnetic potential in both the attraction and repulsion zones was systematically analyzed. From Figure 3 and Figure 4, it is evident that along the *z*-axis intercept line, due to the particle permeability being several orders of magnitude greater than that of air, a strong magnetic flux aggregation occurs at the particle ends. This leads to a highly concentrated and rapidly increasing field intensity gradient at the particle boundary. Within the particle, the magnetic flux density is significantly greater than that in the surrounding air domain, and the magnetic field exhibits quasi-static uniform magnetization characteristics. The relative permeability variation tends toward zero, consistent with the bulk magnetization uniformity predicted by the Maxwell stress tensor theory.

Along the *x*-axis intercept line, the cross-media magnetic field demonstrates the nonlinear evolution behavior of the magnetic field. As the air domain approaches the particle boundary, the magnetic field lines converge, generating a siphon-like effect (Figure 4b). In the air region, as the intercept line nears the particle surface, the magnetic field intensity first weakens; at the particle boundary, it increases stepwise; and within the particle, it ultimately presents a uniformly distributed state.

### 3.2. RVE Magnetostrictive Finite Element Simulation of MREs

In practical applications, MREs exhibit inherent cross-scale characteristics. While the bulk size of MREs typically ranges from several millimeters to hundreds of millimeters, the internal magnetic particles are generally of micron scale [23]. The large disparity in size between the bulk material and its internal microstructure makes it essential to carefully determine the RVE. The selection of an appropriate RVE must account for both the microstructural characteristics and the magnetic field response, and is generally determined through a combination of statistical methods and a convergence analysis.

For spherical particle-reinforced composites such as MREs, Hu Xiaoling [24] reported that, when the ratio of the side length of the RVE to the particle size is below six, the calculation accuracy decreases significantly. When the ratio exceeds six, the influence of the size ratio on the accuracy can be neglected. Based on these findings, this study introduces a statistical convergence analysis method and an intelligent optimization algorithm constructed on a multi-scale Ben-Construction Model, thereby achieving a balance between accuracy and computational efficiency.

In this work, a representative particle mass fraction $\varphi_{m}$ of 50% was selected. The geometric size of the RVE was calculated according to the mass fraction Formula (16).

(16) $$\frac{\frac{4}{3}\pi R^{3}\rho_{1}n}{\frac{4}{3}\pi R^{3}\rho_{1}n+(V-\frac{4}{3}\pi R^{3}n)\rho_{2}}=\varphi_{m}$$

In the formula, $\rho_{1}$ and $\rho_{2}$ represent the density of CIP particles and the matrix, respectively, and *n* represents the number of particles in the RVE. Here, according to the product data of the material supplier, the density of the particles and matrix is $\rho_{1}=7.87\;g/cm^{3}$, $\rho_{2}=1.03\;g/cm^{3}$, and the particle radius is R=2 μm. Through the previous theory and the observation results of the microstructure of MREs, this paper selected *d* / *R* = 2.5, the distance between the two particles was 5 μm, the distance between the particle chains was 7.61 μm, and the matrix material was uniformly filled [23,24,25,26,27,28].

**Figure 5:**
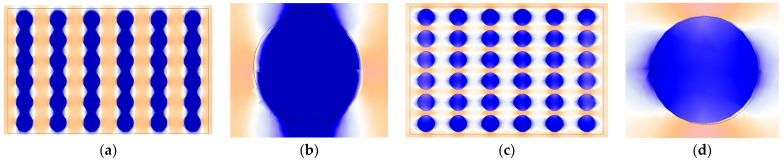
RVE magnetization diagram under different magnetic fields. (**a**) Magnetization diagram of particle chain in z direction magnetic field; (**b**) magnetization diagram of particles in z-direction magnetic field; (**c**) magnetization diagram of particle chain in x direction magnetic field; and (**d**) magnetization diagram of particles in x direction magnetic field.

In the three-dimensional space, the periodic boundary conditions are applied to the RVE to simulate the continuous boundary conditions. Further the uniform magnetic fields are applied along the z-axis direction and the x-axis direction, respectively, and the results are shown in Figure 5. The coexistence of the attraction and repulsion regions will have a considerable effect on the macroscopic properties of the magnetic material (such as magnetostrictive, magnetorheological, and mechanical strength). This is also the reason that MREs exhibit magnetostrictive properties.

As shown in Figure 5a,b, when the magnetic field is applied along the positive *z*-axis, adjacent particles within the chain experience strong attraction along the z-axis due to their close proximity. Consequently, the particles tend to move closer, while the elastomeric matrix constrains the motion. According to the theoretical model in the second section and the three-dimensional elasticity theory of generalized Hooke’s, the component $\varepsilon_{ij}$ of the second-order strain tensor can be expressed as

(17) $$\varepsilon_{ij}=\frac{1+\nu }{E}\sigma_{ij}-\frac{\nu }{E}\sigma_{kk}\delta_{ij}$$

**Figure 6:**
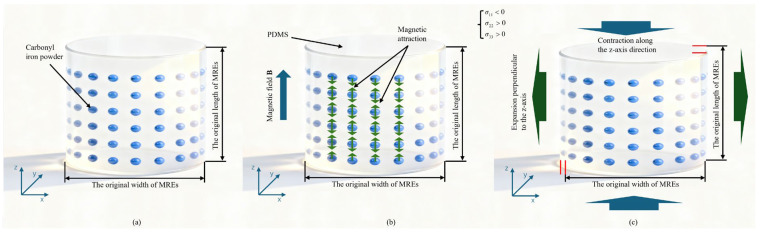
Schematic diagram of magnetostrictive compression characteristics. (**a**) MRE symbolic model; (**b**) application of a magnetic field; and (**c**) magnetostriction phenomenon.

In the formula, $\sigma_{ij}$ is the component of the second-order stress tensor, $\sigma_{kk}$ is the trace of the stress tensor, using the Einstein summation convention, $\delta_{ij}$ is a Kronecker function. In the three-dimensional stress state, the strain-stress relationship of rubber can be described by Equation (17). At this time, the matrix is compressed along the z-axis, and the stress $\sigma_{11}$ in the z-axis direction is negative so that the transverse strains $\varepsilon_{22}$ and $\varepsilon_{33}$ are positive, and the surrounding area of the RVE shows an expansion trend. Equation (17) comprehensively explains the anisotropic deformation behavior of the matrix during compression from the perspective of macroscopic continuum mechanics to microscopic molecular statistics, as shown in Figure 6.

**Figure 7:**
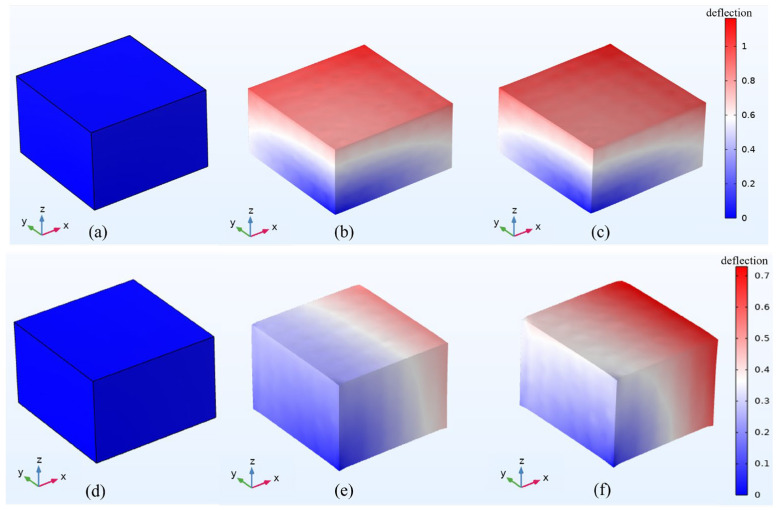
Magnetostrictive deformation diagram of RVE under different magnetic fields. (**a**) RVE deformation diagram of 0.1 T magnetic field in z direction; (**b**) RVE deformation diagram of 0.6 T magnetic field in z direction; (**c**) RVE deformation diagram of 0.9 T magnetic field in z direction; (**d**) RVE deformation diagram of 0.1 T magnetic field in x direction; (**e**) RVE deformation diagram of 0.6 T magnetic field in x direction; and (**f**) RVE deformation diagram of 0.9 T magnetic field in x direction.

As shown in Figure 7a–c, the magnetostrictive compression phenomenon of the RVE indicates that as the magnetic field continues to increase, the magnetostrictive amount of the RVE tends to increase. However, the difference in deformation between the 0.6 T and 0.9 T magnetic fields is small, and the magnetostrictive deformation may have progressive saturation characteristics with and increasing magnetic field. This phenomenon is closely related to microscopic mechanisms such as matrix constraint field reconstruction and the magnetic particle chain stretching phase transition.

As shown in Figure 7d–f, when the magnetic field is applied along the positive x-axis, adjacent particles in the chain experience a repulsive force. According to Equation (17), this results in elongation along the *x*-axis, while the upper and lower boundaries of the RVE show contraction. Overall, the RVE exhibits macroscopic magnetostrictive elongation. Figure 7d–f illustrate that, when fields of 0.1 T, 0.6 T, and 0.9 T are applied, the elongation of the RVE increases progressively with increasing magnetic field strength.

**Figure 8:**
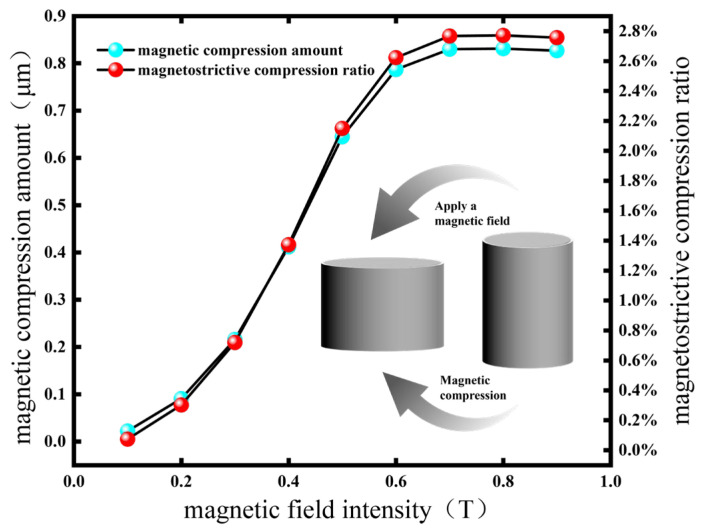
The curve of magnetostrictive compression effect with magnetic field intensity.

When the external magnetic field is parallel to the particle chain direction, in the initial stage, the field intensity lies within the initial magnetization region of CIP particles. In this region, the particle magnetization increases linearly with the applied field intensity. Consequently, the compression of the RVE increases rapidly, and the magnetostrictive compression rate grows nonlinearly. At 0.6 T, the compression rate of the RVE reaches 2.62%. When the field strength exceeds 0.6 T, the particles enter the saturated magnetization zone, the growth rate of magnetization slows, and the strain growth rate decreases from 4.7%/T to 1.5%/T, marking the onset of the deformation saturation transition zone. At this stage, the inter-particle magnetic dipole force increases only slightly, resulting in limited additional strain. At 0.7 T, the magnetostrictive compression rate reaches 2.77%. With further increases in the field strength, particle magnetization approaches the saturation magnetization $M_{s}$, and RVE deformation also saturates. The orientation of the particle chain tends toward complete ordering, and the increment of magnetic strain approaches zero. As shown in Figure 8, the deformation is about 0.83 μm, and the compression rate stabilizes at 2.77%.

Because PDMS is an elastic material, the storage modulus $G^{\prime }$ of the matrix resists deformation through the strain energy density $U=G^{\prime }\gamma^{2}/2$, and reaches saturation when Equation (18) is satisfied.

(18) $$F_{\max}=G^{\prime }\gamma_{sat}$$

**Figure 9:**
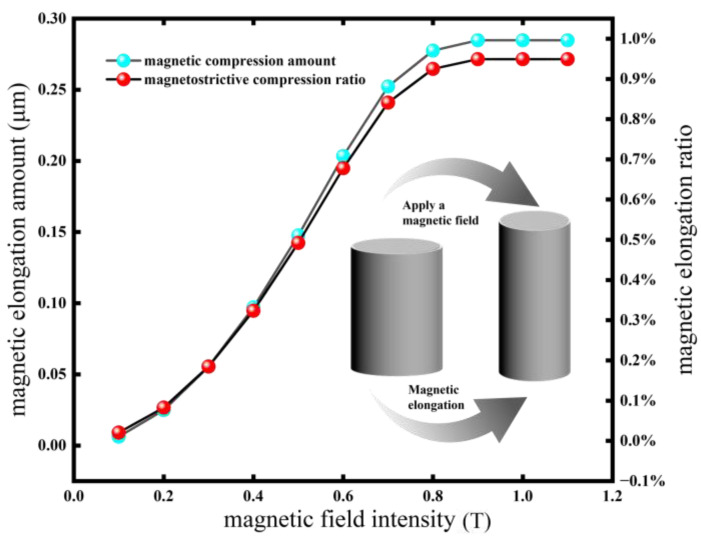
The curve of the magnetostrictive elongation effect with magnetic field intensity.

In the formula, $F_{\max}$ is the interaction force between saturated magnetic dipoles, and $\gamma_{\mathrm{sat}}$ is the saturated strain. Continuing to increase the magnetic field strength leads to an increase in hysteresis loss (increase in energy consumption), and the deformation of the RVE does not increase. The magnetostrictive study of MREs has little practical significance, so this paper does not discuss it. When the direction of the external magnetic field is perpendicular to the direction of the particle chain, it is the same as the above magnetostrictive compression, but the interaction force between the two particles inside the particle chain is a repulsive force. When the magnetic field reaches 0.8 T, the maximum magnetostrictive elongation of the RVE is approximately 0.27 μm, as shown in Figure 9, and the elongation can reach 0.95%.

### 3.3. Study on the Magnetostrictive Properties of RVE with Non-Ideal Particle Chain Structure

The preparation of anisotropic MREs is a multi-physical field coupling process. During fabrication, magnetic particles are subjected to viscous dissipation forces, rheological effects, dipole–dipole interactions, and thermodynamic disturbances of the matrix. These nonlinear interactions result in particle chains exhibiting a statistically short-range order rather than an ideal linear alignment. This phenomenon can be quantitatively described using orientation order parameters derived from Langevin dynamics simulations.

**Figure 10:**
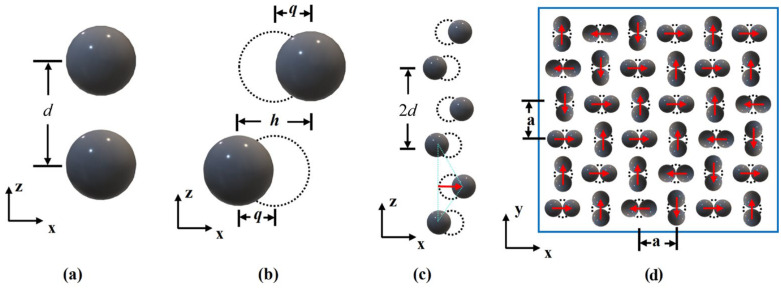
Modeling process of various particle chain linearities of the RVE. (**a**) Create two spheres with center distance *d* along z-axis direction; (**b**) The two spheres are moved along the x-axis in both positive and negative directions; (**c**) The spheres in (**b**) are arrayed along the positive direction of the z-axis; (**d**) The particle chain array is rotated 90° in turn along the original center line.

To replicate the actual chain formation process, this study first took two adjacent particles at the end of the particle chain (negative direction of the z-axis), as shown in Figure 10a. The two particles were translated *q*μm along the positive and negative directions of the x-axis (Figure 10b, *h* = 2*q*). The protrusion height *q* was used to characterize particle chains with varying degrees of linearity. Taking the two particles after the translation transformation in Figure 10b as the object, the two particles were arrayed along the positive direction of the z-axis to obtain the particle chain structure shown in Figure 10c. The particle chains were evenly and equidistantly distributed in the matrix material. Figure 10d shows the top view of the rotating particle chain of the RVE model, and the protrusions are pointed to, as shown by the arrows in the figure.

**Figure 11:**
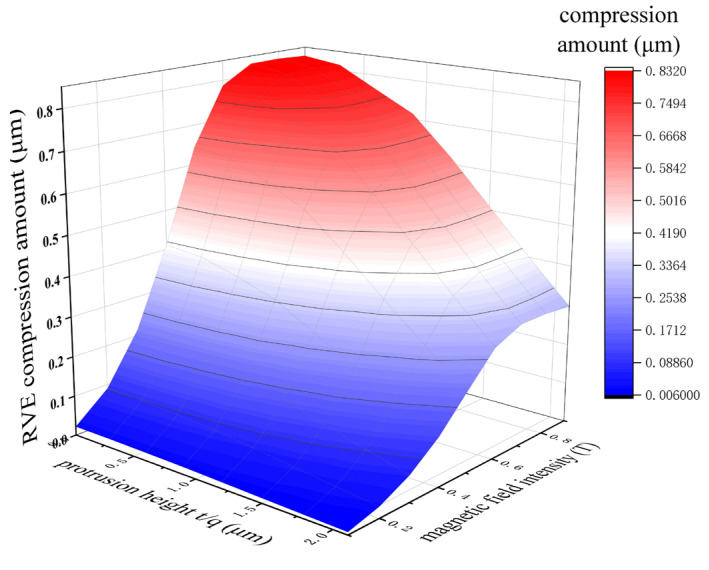
Magnetostrictive compression of RVE under magnetic field.

**Figure 12:**
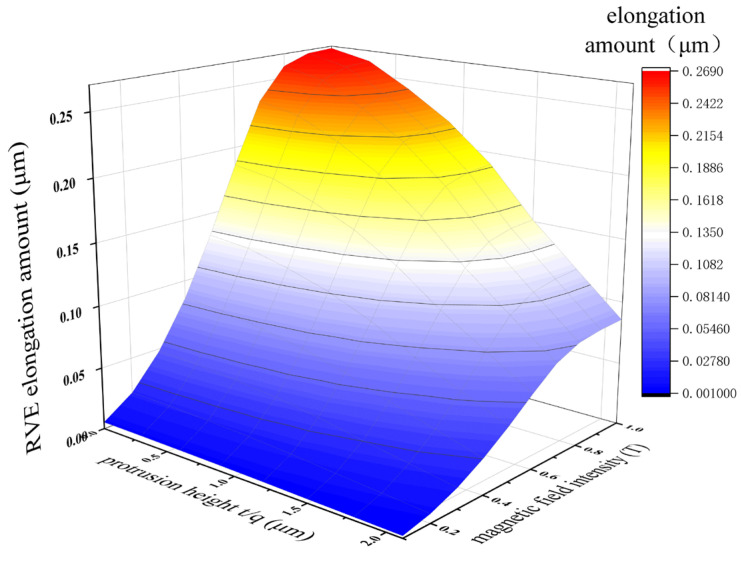
Magnetostrictive elongation of RVE under magnetic field.

Through the calculation of the above model, the results are shown in Figure 11 and Figure 12. In Figure 11, it can be seen that the magnetostrictive compression characteristics were significantly enhanced with an increase in the linearity of the particle chain and the magnetic field strength. When the linearity was 100% (that is, the particle chain was an ideal straight line) and the magnetic field strength reached 0.7 T, the magnetostrictive compression characteristics of the RVE reached a maximum of 0.83 μm, and the subsequent compression will not continue to increase with an enhancement in the magnetic field. In Figure 12, the change trend in magnetostrictive elongation characteristics is the same as that of magnetostrictive compression characteristics. When the magnetic field reaches 0.8 T, the magnetostrictive elongation reaches the maximum value, which is about 0.27 μm.

With decreasing linearity of the particle chain, the magnetostrictive properties of MREs are weakened in terms of the dimensions of particle magnetization, magnetic dipole interactions, anisotropy, stress transfer and energy dissipation [25]. As shown in Figure 11 and Figure 12, with decreasing linearity, the magnetostrictive compression and elongation properties of the RVE gradually weaken, whereas the saturation magnetostrictive magnetic field strength gradually increases. When the linearity is 100%, that is, when the particle chain is an ideal straight line, the magnetostrictive properties of the RVE are the best, which is completely consistent with the results described by the above theoretical model.

## 4. MRE Comprehensive Test

### 4.1. Preparation Test of MREs

The preparation of MREs used two core materials of CIP and PDMS silicone rubber. The core parameters of the materials selected in this experiment are shown in Table 1, Table 2 and Table 3.

**Figure 13:**
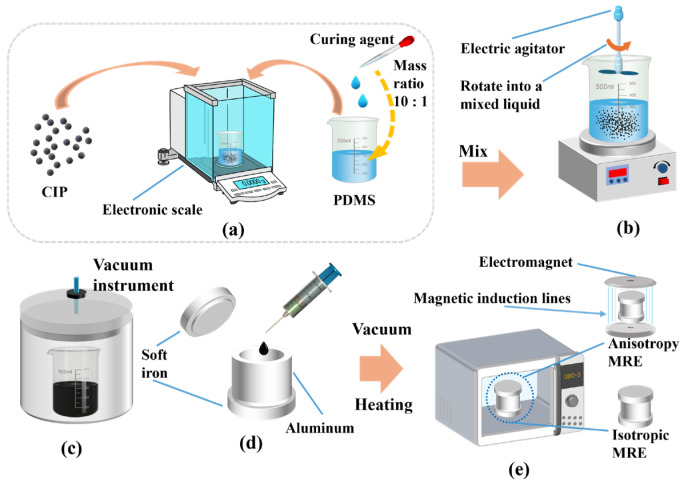
Preparation process of MREs. (**a**) Weighing ratio; (**b**) mixing; (**c**) vacuum treatment; (**d**) injection mould; (**e**) curing process.

The preparation process of MREs can be summarized into five key stages: weighing, mixing, molding and curing (Figure 13). The mixture of carbonyl iron powder particles (China Metallurgical Research Institute, Hebei, China) and the same mass of polydimethylsiloxane mixture (PDMS, Dow Corning Corporation, Midland, MI, USA; the mixture here refers to the mixture of PDMS and curing agent at a mass ratio of 10:1) was fully stirred in a stirrer. The mold was then placed in a vacuum oven at 60 °C for 4 h. The vacuum step removed bubbles formed during mixing, ensuring uniform curing. MREs with different internal structures were prepared by applying solidified magnetic fields during curing [26,27,28,29].

**Figure 14:**
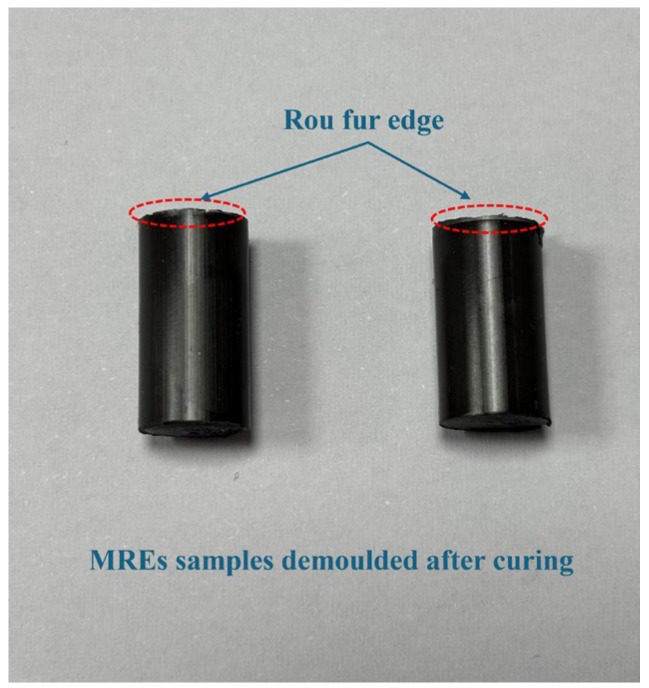
Finished MRE prepared.

After the preparation process shown in Figure 13, the finished sample of MREs is shown in Figure 14. In this experiment, MRE samples of 0 mT, 50 mT, 100 mT, 150 mT and 250 mT were prepared. At this time, there were some burrs on the upper and lower ends of the finished product, which were put into use after smooth processing.

### 4.2. MRE Observational Experiments

**Figure 15:**
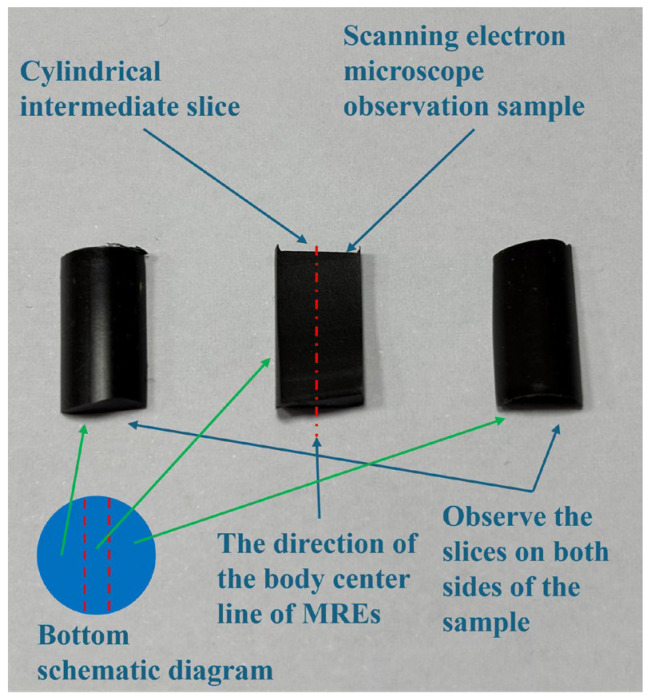
Observation sample of MREs.

In order to study the distribution state of the internal particle chain of the MRE samples, the MRE finished products obtained under different solidified magnetic fields were observed by SEM. The observed samples are shown in Figure 15, and a cross-section of the observed samples was observed. Since MREs have a good electrical conductivity, there was no need to apply a conductive layer in the test.

**Figure 16:**
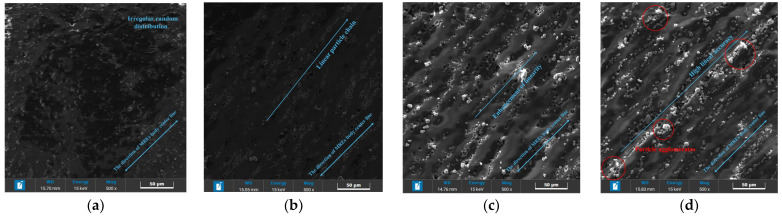
Scanning electron microscope image of MREs. (**a**) Solidified magnetic field, 0 mT; (**b**) solidified magnetic field, 50 mT; (**c**) solidified magnetic field, 150 mT; and (**d**) solidified magnetic field, 250 mT.

In the absence of a solidified magnetic field, internal particles are randomly distributed, forming isotropic MREs, as shown in Figure 16a. Under solidified magnetic fields, the particle chains achieve varying degrees of linearity. With an increase in the intensity of the solidified magnetic field, the linearity of the particle chain is gradually improved, and the degree of particle polymerization is also gradually improved. When the solidified magnetic field is 150 mT, it can be observed that the polymer is formed between the particles. When the intensity of the solidified magnetic field reaches 250 mT, the internal particle polymer can be clearly observed.

### 4.3. Experimental Measurement of Magnetostrictive Properties

**Figure 17:**
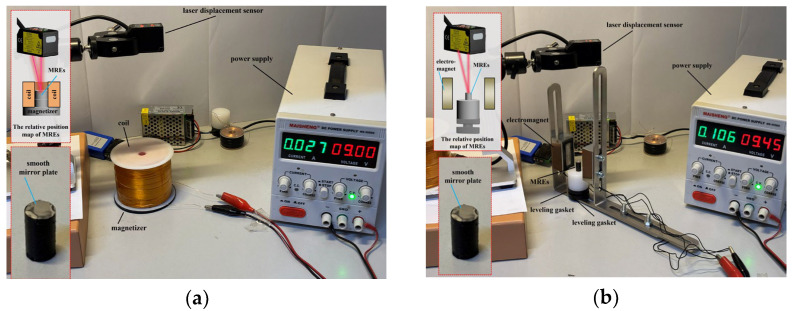
Magnetostrictive characteristic observation test. (**a**) Coil magnetostrictive compression characteristic test bench; (**b**) magnetostrictive elongation characteristic test bench.

To investigate the magnetostrictive properties of various MREs under applied magnetic fields, a test bench was constructed using high-precision laser displacement sensors, electromagnetic coils, and electromagnet instruments (Figure 17). The electromagnetic coil was used to provide a magnetic field parallel to the particle chain inside the MREs (Figure 17a), while the electromagnet was employed to apply a field perpendicular to the chain direction (Figure 17b). The field strength was controlled through the power supply. At the same time, an ultra-thin smooth mirror patch was pasted on the surface of the MREs to further improve the accuracy of the test measurement.

**Figure 18:**
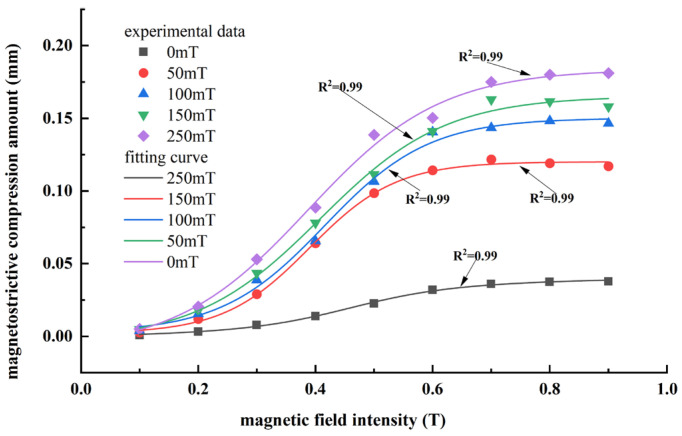
Experimental curve of magnetically induced magnetostrictive compression characteristics of MREs.

**Figure 19:**
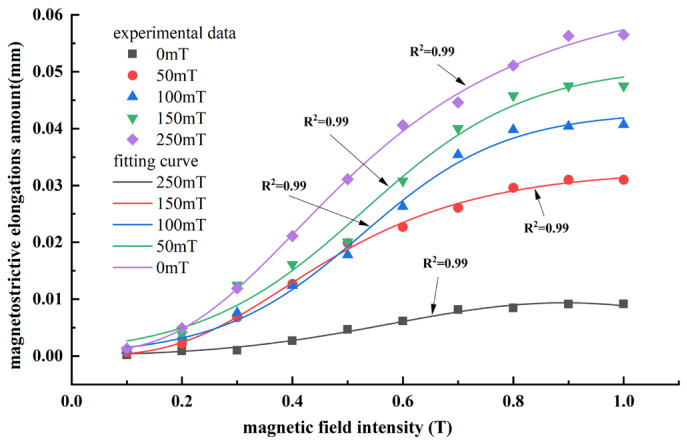
Experimental curve of magnetically induced magnetostrictive elongation characteristics of MREs.

The test samples were cylindrical MREs with a bottom radius of 4 mm and a height of 10 mm, with the internal particle chains aligned along the cylinder axis. Due to the complexity of the internal particle chain morphology of MREs, it is difficult to use the determined parameters for a quantitative analysis. In this paper, MREs formed under different magnetic field strengths were tested to indirectly characterize the influence of different particle chain linearities on their magnetostrictive properties. It includes an isotropic MRE with a solidified magnetic field of 0 mT and four anisotropic MREs with solidified magnetic fields of 50 to 250 mT. The point results shown in Figure 18 and Figure 19 were obtained by the test, and the curve shown in Figure 18 and Figure 19 was obtained by curve-fitting of the test results.

From Figure 18 and Figure 19, it was observed that the experimental results are in overall agreement with the numerical simulations, following the same trend. However, the experiments clearly show that the magnetostrictive performance of isotropic MREs is much lower than that of anisotropic MREs. For anisotropic MREs prepared under a solidified magnetic field of 250 mT, the maximum measured magnetostrictive compression ratio was 1.81%, while the maximum magnetostrictive elongation reached 0.565% at an applied magnetic field of about 1.0 T.

The significant difference between the experimental results and the maximum magnetostrictive effect presented by the numerical simulation can be mainly attributed to two reasons: (i) during fabrication, the linearity of the internal particle chains cannot achieve the ideal state, resulting in deviations from equidistant particle distribution and the occurrence of particle agglomeration, and (ii) the magnetic field applied during experiments cannot achieve perfect uniformity, leading to additional deviations.

## 5. Conclusions

Based on the modified magnetic dipole theory, the interaction between magnetic particles under an applied field was analyzed at the mesoscopic scale, and a theoretical model incorporating particle chain linearity was developed. By varying the direction and intensity of magnetic field loading and the degree of particle chain linearity, the magneto-mechanical mechanisms underlying different magnetostrictive behaviors of MREs were deduced. MREs with different particle chain linearities were fabricated and subjected to magnetostrictive testing, and the experimental results were consistent with theoretical predictions and numerical simulations. The main conclusions are as follows:

(1) The magnetostrictive compression and elongation characteristics of MREs depend on the relative angle between the applied field direction and the particle chain orientation. When the magnetic field is parallel to the particle chain, MREs exhibit magnetostrictive compression. When the field is perpendicular, they exhibit magnetostrictive elongation.
(2) The linearity of the particle chain in MREs is influenced by the strength of the solidified magnetic field. Within a certain range, chain linearity increases with the field strength. However, when the field reaches a sufficiently high intensity, particle agglomeration occurs.
(3) The magnetostrictive properties of MREs exhibit saturation. Under the conditions of this study, the maximum theoretical and experimental magnetostrictive compression rates reached 2.77% and 1.81%, respectively, while the maximum theoretical and experimental elongation rates reached 0.9% and 0.565%, respectively. Compression was found to be more pronounced than elongation.
(4) The magnetostrictive properties of MREs are strongly dependent on particle chain linearity. A higher chain linearity results in a stronger magnetostrictive performance, and when the chain linearity approaches the ideal value of 100%, the magnetostrictive properties reach the ideal state.

## Figures and Tables

**Table 1:** Physical properties of PDMS when supplied.

Physical Properties	Numerical Value	Unit
Viscosity, 23 °C (main agent)	5500	mPa·S
Viscosity of PDMS after mixing with curing agent at 23 °C	4000	mPa·s

**Table 2:** Physical properties of PDMS cured at 60 °C for 4 h.

Physical Properties	Numerical Value	Unit
Young’s modulus	1.6	MPa
Poisson ratio	0.495	
Relative permeability	1	
Working temperature	−50~+200	°C

**Table 3:** Physical properties of carbonyl iron powder.

Physical Properties	Numerical Value	Unit
Density	7.87	g/cm^3^
Average particle size	4	μm
Young’s modulus	210	GPa
Poisson ratio	0.3	
Relative permeability	3000	

## Data Availability

The original contributions presented in this study are included in the article. Further inquiries can be directed to the corresponding author.
